# Dislocation of the Humerus on the Dorsum of the Scapula; Reduction

**Published:** 1851-09

**Authors:** 


*Dislocation of the Humerus on the Dorsum of the Scapula; Reduction.* (Under the care of Mr. Stanley.)—The nomenclature of the various dislocations of the shoulder has given rise (at least among surgeons) to frequent discussions, which, as is too often the case, have not ended in a perfect understanding upon the subject; and the English reader, after taking cognizance of the divisions partially adopted in France and Germany, gladly returns to the denominations, *downwards*, *forwards*, and *backwards*, taught in our schools. The luxation which we have now to consider, and which happened in a patient of Mr. Stanley's, is called by some French surgeons the sub-variety of the sub-acromial, and by others, sub-spinal; the Germans, as stated by Mr. Smith in "Chelius' Surgery," call it outwards; but most surgeons will agree with us, that backwards, or on the dorsum scapulæ, is as correct a name as can be desired. That this displacement occurs but very seldom is a well known fact; it had not been seen for a long period at St. Bartholomew's Hospital; and Sir Astley Cooper says, in his work on Dislocations and Fractures: "Two cases only of this accident occurred in Guy's Hospital in thirty-eight years, the first during my apprenticeship. The nature of the accident was at once obvious from the projection of the head of the bone on the dorsum scapula. The bandages were applied in the same manner as if the head of the humerus had been in the axilla, and the extension was made in the same direction as in that accident." The reduction was easily effected; the second case presented itself several years afterwards, and was reduced by the dressers. Sir A. Cooper mentions seven more cases, which mostly occurred in private practice.

That the reduction took place so readily, by using the same means as for dislocation into the axilla, might create surprise, for it seems at first sight natural that extension should be made a little outwards, when the head of the bone is lodged on the dorsum of the scapula. But the dislocations of the shoulder-joint are generally fraught with so little danger, the reduction is commonly so easily obtained by proper extension and coaptation, that it would be, perhaps, unwise to establish very nice distinctions as to the precise direction in which extension is to be made.

Mr. Stanley's patient was a farm laborer, aged sixty-two years, a strong, healthy-looking man, who was admitted into St. Bartholomew's Hospital, on the 4th of April, 1851, suffering from a dislocation of the shoulder-joint. The patient states that he was driving in a cart, in the company of a fellow-laborer when the horse fell, and both of the men were thrown or jerked out of the cart. One of them escaped unhurt, but the patient was violently thrown on his right shoulder, the whole weight of the fall being borne by that articulation. The contusion and laceration were not considerable, as he luckily fell on a plot of grass; but it would appear that the lesion was of a serious character, as some of the actions of the joint were suddenly lost.

The patient applied at once to a surgeon, who told him that the limb was dislocated, and attempted reduction, by putting his heel into the patient's axilla, whilst the latter was lying on his back. The efforts were, however, fruitless, and the man was advised to repair to this hospital. On examination, it was found that the axis of the right humerus, in its relation to the body, was very much altered. Beneath the spine of the scapula, and towards its outer side, was a firm hard swelling: this was the head of the bone, the fact being proved negatively by its absence in the proper position, and positively by the swelling moving when the arm was being rotated. The glenoid cavity can be felt empty; there is very little motion of the arm, and the deltoid is tense. On rotating the arm, a kind of crepitus is distinguished, which does not feel like the ordinary crepitus of fracture, but is probably occasioned by the rubbing of the head of the bone against the axillary border of the scapula.

Mr. Stanley having determined to proceed at once to the reduction of the limb, the man was placed in a chair, and his body fixed; extension was first made in a direction outwards and rather downwards, the limb being grasped above the elbow. Scarcely had the extension begun to be used, before the bone was felt to slip from the scapula into the axilla. Further extension was made for about ten minutes, with no further effect. A jack-towel was now placed around the upper part of the humerus, and the head drawn upwards; extension was then suddenly remitted, and the bone slipped into its socket. After a short stay in the hospital, and proper confinement of the shoulder, the patient was discharged with full use of his arm.—*Lancet, 12th July* 1851.

